# Digitalisation of occupations—Developing an indicator based on digital skill requirements

**DOI:** 10.1371/journal.pone.0278281

**Published:** 2023-01-17

**Authors:** Carolina Lennon, Laura Samantha Zilian, Stella Sophie Zilian

**Affiliations:** 1 Graz Schumpeter Centre, University of Graz, Graz, Austria; 2 Department of Economics, Vienna University of Economics and Business, Vienna, Austria; King Abdulaziz University, SAUDI ARABIA

## Abstract

Digitalisation is assumed to have far reaching consequences for workers. So far, these have been analysed using indicators derived from survey data on occupational tasks. Survey-based indicators measure what people do at work but provide little insight into the skills required to perform a task. Since multiple skills may be necessary to perform a task, approximating digital skills through tasks may underestimate the extent of digitalisation of a given occupation. Besides, they provide limited coverage in terms of periodicity, scope and variety of tasks. We therefore suggest to change the perspective from tasks to skills and propose to analyse the digital skill requirements of occupations. To this end, we use detailed information on the classification of European Occupations, Skills and Qualifications, natural language processing tools and network analysis methods to determine digital skills in the database. We construct four different versions of the digital competencies indicator identifying occupations that depend highly on digital skills. Our indicator can be mapped to the ISCO-08 classification and easily be used alongside other data sources. We show that compared to an indicator based on ICT-tasks derived from the OECD ‘Programme for the Assessment of Adult Skills’, our indicator captures more complex and specialised digitalised occupations. Our results stress the importance of using granular data in order to properly identify digital skill requirements of jobs.

## Introduction

Digitalisation transforms every realm of our society and, in particular, for workplaces, this process has been accelerated by the on-going Covid-19 pandemic. This digital transformation, however, is not limited to specific IT-jobs or industries, but affects the whole world of work. Several studies forecast a replacement of jobs by digital technologies ranging between less than 10 per cent up to more than 50 per cent (e.g. [1–3]). While these studies are future-oriented, the work in [4, 5] shows that these changes are already taking place in the US labour market. Similarly, the results of [6] suggest that employment in jobs related to digital tasks tends to grow faster in Italy. Overall, these studies indicate that the widespread use of ICT at work changes skill requirements of jobs, and that the demand for ICT-related skills is expected to continue to rise thereby affecting wage differentials between workers with different levels of digital skills [7].

Consequently, measuring the digital skill requirements of occupations is highly relevant to inform research and policy makers in the face of societal challenges posed by the digital transformation. With this paper, we therefore aim to provide an indicator to measure the degree of digitalisation based on digital skills using the classification of European Skills, Competences, Qualifications and Occupations (ESCO).

Thus far, several indicators have been developed to quantify ICT skill requirements of jobs. These indicators typically approximate skills via tasks based on data collected through surveys assessing what workers do at work. Among the surveys most commonly used, we find the OECD ‘Programme for the Assessment of Adult Skills’ (PIAAC) [8, 9], the Italian ‘Indagine Campionaria sulle Professioni’ (ICP) [6], and the survey data from US Occupational database ONET [4].

Occupational indicators derived from survey data, however, suffer from some drawbacks: self-reported tasks are affected by respondents’ perception of an occupation and they are therefore subject to survey bias. Most surveys are not updated frequently and are therefore ill-fitted to track changing task compositions. Moreover, surveys cannot go into great detail on the variety and specificity of tasks. Finally, while tasks measure what people do at work, they provide little information on the skills required to perform a task. Given that multiple skills may be necessary to perform a task [10], approximating digital skills through ICT tasks alone, may therefore underestimate the extent of digitalisation of a given occupation. Thus, despite the popularity of task-based approaches to estimate labour market effects of computerisation (e.g. [11–14]), they might not be able to adequately capture nuanced variations in the degree of digitalisation of occupations.

To overcome the above mentioned drawbacks, digital skills indicators can alternatively be constructed based on dictionaries of occupations, which map tasks, skills, and knowledge requirements to occupations. Examples of such dictionaries are provided under the ESCO framework and as a part of the ONET database. Thus far, they have been relatively underutilised for measuring digital skills requirements of occupations in the economic literature. This is surprising as dictionaries of occupations offer numerous advantages. In particular, the ESCO database, besides being of free-access, offers an unprecedented level of granularity, currently covering more than 3,000 occupations—three times more than the number of occupations in the ONET database—and almost 14,000 skills and competencies. Moreover, the database is regularly being revised, and the set of digital skills covered in the database are constantly updated [15]. Finally, the ESCO database adheres to the latest version of the International Standard Classification of Occupations (ISCO-08). This is a clear advantage over the ONET database, which is based on the Standard Occupational Classification of the USA (SOC). This ensures that indicators based on the ESCO database can be readily applied to a variety of surveys without loosing information from the conversion between occupational classifications.

To the best of our knowledge, only the work in [16] employs the ESCO classification to construct a set of digital skills indicators. In this paper, we expand on the work presented in [16] by tapping into the wealth of information currently offered by the ESCO classification. We construct a set of Digital Competencies Indicators (DCI) inspired by network analysis methods and using Natural Language Processing (NLP) tools. Compared to the work in [16] our methods are able to identify 16 times more digital skills. Moreover, while the authors in [16] construct indicators only for nine groups of the 1-digit ISCO classification, we construct and make available our indicators at the lowest level of the hierarchy of the ESCO classification, which can then be aggregated at any level of the ESCO and ISCO classification.

Finally, we also analyse how our indicator performs in relation to indicators based on survey data on tasks at work. To this end, we construct a task-based indicator using the PIAAC survey, which has been widely used in the literature. Compared to the task-based indicator, our indicator fares better at identifying different degrees of digital specialisation of occupations, while the task-based indicator captures a more generic type of ICT tasks that are used across many occupations. We also find that the tasks indicator seems to be relatively more aligned to the skill level of occupations, which is typically approximated by the ISCO major groups. This suggests in turn that our indicator might be better at conveying information on digital skill requirements that goes beyond this broad classification.

Regarding our indicator based on the ESCO database, we find a large dispersion of the value of the indicator within occupational groups. This, on the one hand, highlights the importance of using granular data in order to properly identify digital skill requirements of jobs, and thus, plays in favour of the use of a dictionary of occupations for the construction of digital skills indicators. On the other hand, this suggests that, whenever there is an opportunity, the indicator should be applied at the lowest possible level of the occupational hierarchy.

The remainder of the paper is structured as follows. In the literature review, we discuss existing indicators measuring the degree of digitalisation of occupations. We then present the ESCO database and explain the methods to derive a digital competencies indicator based on skill requirements of jobs. Next, the results on differences between occupations regarding the degree of digitalisation are presented. Finally, we compare the DCI to an indicator derived from survey data provided by PIAAC. The last section concludes.

## Literature review

The PIAAC survey is frequently used to construct task-based indicators to determine what people do at work. Even though the focus of these task-based indicators is not primarily on digital tasks, sub-indices of these composite indicators have been used to investigate the impact of new technologies on work [17] and to identify the share of ICT task-intensive occupations in OECD countries [18]. Hence, in the following paragraph we will summarise how these (sub-)indicators are derived and what findings related to digital tasks were obtained.

One sub-indicator based on PIAAC is developed by [8], whose original goal was to characterise the distribution of skills across countries, industries and jobs. Furthermore, they try to identify the skills that are important for individual’s performance on the job as well as for firm performance and how the skill endowments of the workforce and the frequency of job tasks performed relate to firms’ participation in global value chains. For their analysis, they use data obtained by the PIAAC assessment on workers’ self-reported tasks to approximate for skills possessed by individuals. Based on 57 PIAAC survey items, the authors conduct a exploratory factor analysis and construct six task-based skills indicators, i.e. information and communication technologies (ICT) skills; readiness to learn and creative problem solving; managing and communication; self-organisation; marketing and accounting; and science, technology, engineering and mathematics (STEM)—quantitative skills.

One of these indicators, namely, information and communication technologies (ICT) skills, was used to define ICT task-intensive occupations on the ISCO-08 3-digit level in [18, p.122]. According to [18], the share of workers in ICT task-intensive occupations increased from 2011 until 2017 in almost all OECD countries, ranging from 5 per cent in Turkey up to 20 per cent in Luxembourg in 2017. Additionally, together with ICT specialists, ICT task-intensive occupations contributed positively to employment growth in almost all OECD countries.

Another sub-indicator related to digital tasks was developed by [9] who used the PIAAC survey alongside the European Working Conditions Survey (EWCS) and the US occupational database ONET to construct different task indices in order to gain insights into what people do at work in Europe. Their task framework covers physical tasks, intellectual tasks, social tasks, work organisation, and technology, where the latter encompasses not just the use of computers, but also the use of non-ICT machinery. Based on PIAAC, the authors also distinguish between basic ICT tasks and programming ICT tasks for the subindex ‘Operation of IT’. Their analysis of the technology task indices on the ISCO-08 2-digit level shows that agricultural and industrial (non-professional) occupations are significantly less technology-intensive than the rest. Regarding the sub-indices based on PIAAC, they further highlight that basic ICT tasks are important in managerial, professional, administrative and service occupations, whereas advanced programming ICT tasks only matter in very specific occupations, such as ‘Information and communication technician professionals’.

More recently, [19] released a new version of the task framework mentioned above, which is based on a revised version of the taxonomy presented in [20] where several new concepts and components are added. Most importantly, they replace the ONET occupational database with the Italian occupational database ‘Indagine Campionaria sulle Professioni’ (ICP) provided by INAPP-ISTAT and use a more recent version of the EWCS (2015). In the current version, the ICT sub-indices are based on information from ICP and PIAAC.

An application of the ICP survey to the construction of digital competencies indicators is also found in [6]. In their paper, the authors investigate the relationship between employment growth and jobs’ intensity in routine and digital tasks in Italy between 2011 and 2016. To identify jobs that rely intensively on routine and digital tasks, they employ the most recent release of the ICP survey, which already dates back to 2012. Besides constructing an indicator to account for the routine task-intensity of jobs following a similar methodology as in [14], the authors also construct two indicators to account for workers’ use of digital technologies. Their first digital indicator is based on two questions of the survey in which workers are asked about the frequency in which they use emails and the frequency and complexity of tasks they perform involving computers and information systems. For the second digital indicator, they use a free-form question where respondents are asked to list the main 15 tasks performed at work as well as their relative importance. Based on this information the authors identify distinct words mentioned in workers’ answers and manually flag those relating to digital technologies. As such, from a total of 5,700 distinct words, they identify 51 that explicitly refer to digital technologies and that were mentioned in 131 tasks reported by workers. Merging their indicators to the ISTAT Italian Labour Force Survey, they find that employment in jobs intensive in digital tasks tends to grow faster.

Other examples of how surveys have been used to identify occupations that rely on digital technologies are [4, 5]. While the former uses data collected for the US occupational database ONET, the latter developed a survey (STAMP) to capture direct information on US jobs characteristics such as e.g. jobs’ skill requirements or technology use. The STAMP survey was conducted between 2002 and 2006 (first wave) and between 2007 and 2009 (second wave) among employed wage and salary workers in the US at the age of 18 and over. In total, 166 items related to job characteristics were collected and out of these, 27 questions were related to computer use. More specifically, the respondents were asked questions regarding the use of computers, particular applications and functions, and the levels of task complexity, as well as their user competence. The results of the second wave show that ICTs are widely used, as about 70 per cent of respondents reported using a computer at least a few times per week, but the level of complexity of these technologies is low to moderate. For instance, while 40 per cent of all workers use spreadsheet software, only 12 per cent use more complex functions such as macros or equations.

On the other hand, [4] analyse the digitalisation of the American workforce using survey data provided by ONET, which is conducted on a regular basis among a sample of job incumbents and job experts. Each worker is asked to complete one of three questionnaires, each covering a different topic area, namely, ‘General Work Activities’, ‘Knowledge’ and ‘Work Context’. The ‘General Work Activities’ questionnaire provides two technology-related variables (‘knowledge—computer and electronics’ and ‘work activity—interacting with computers’), which [4] use to derive occupation-specific digital scores. Based on these digital scores they determine three tiers of digitalisation of occupations (low, medium, high). Similar to [18], they find that from 2002 until 2016, employment shares and total employment increased in occupations with high and medium digital scores.

Finally, some limitations of the survey-based indicators to measure the degree of digitalisation of occupations must be mentioned: (i) self-reported tasks are subject to workers’ perception of an occupation, and like any self-reported question, they are subject to survey bias as “research finds incumbents generally give their jobs more positive ratings than job analysts or other external observers, such as supervisors” [5, p.180]. (ii) To assess changes in workers’ task composition, surveys need to be conducted regularly. Especially if the focus of the analysis is related to digital technologies, which evolve at a rapid pace, the survey results may be outdated, since skills required to perform a job ten years ago, might not be relevant today. Additionally, the emergence of new technologies may generate new skill requirements that have to be accounted for in the survey questionnaires [15]. Given that PIAAC, STAMP, and ICP were conducted (more than) ten years ago, this limitation is relevant for all indicators based on these surveys. (iii) Due to time constraints only a limited number of task items can be listed in a survey, hence, variations of tasks within and between occupations might not be sufficiently captured. For instance, the number of survey items used in the presented literature ranges from 2 survey items (by [4]) to 27 survey items (in the STAMP survey). (iv) Last but not least, task-based indicators may not adequately capture the related skills required to perform these digital tasks. Even though “tasks are what people do at work, and the introduction of a new technology at work will generally change (remove, transform or add) specific types of task content” [20, p.18], multiple skills may be necessary to perform one task and each job may require a variety of competencies [10, p.5].

Digital skills indicators can alternatively be constructed based on dictionaries of occupations. These dictionaries map tasks, skills, and knowledge requirements to occupations. Probably the most well-known example of such dictionaries is the former Dictionary of Occupational Titles (DOT) in the USA that evolved into the ONET today. More recently in 2017, the European Commission also launched its own dictionary of occupations under the ESCO framework. Although the ONET database has been extensively used for the construction of indicators measuring the routine task content of jobs, we find very few examples in which dictionaries of occupations have been specifically used to measure digital skill requirements of jobs.

To the best of our knowledge, only the work in [16] provides an example on how the ESCO classification can be used to identify digital skills in economic analyses. In their paper, the authors investigate whether the endowment of workers with digital skills might spur diversification towards new technologies across regions in Europe. To assess the digital skills endowments of workers, they first construct a digital skills indicator based on the first version of the ESCO classification. In particular, they identify digital skills in the ESCO database as skills whose label contains the terms ‘computer’ or ‘technolog*’, finding a total of 69 digital skills with this search. They then manually classify the digital skills into three categories depending on the level of professional expertise involved, from the user level at the lowest end to developers level at the highest end. Based on this information, they then construct four indicators; one for each level of expertise and one encompassing all digital skills.

## A digital competencies indicator based on ESCO

Tapping into the wealth of information currently offered by the ESCO classification, we propose the construction of a Digital Competencies Indicator (DCI) that expands on the indicator presented in [16]. First, our method is able to identify a larger set of digital skills. More precisely, our approach identifies 1,151 digital skills, 16 times as many as the digital skills found in [16]. Moreover, while in [16] the authors construct their indicators for only nine groups of the 1-digit ISCO classification, we construct and make available our indicators at the lowest level of the hierarchy of the ESCO classification, which currently consists of more than 3,000 occupations and which can be matched one-to-one to the lowest level of the hierarchy of the ISCO classification.

Despite being relatively new, the ESCO database has been updated constantly. More importantly, the ESCO database continually revises the set of digital skills covered in the framework so that digital skills requirements can better reflect the current reality in the labour market. The research in [15] suggests that the ESCO database can indeed represent a valuable, up-to-date tool to identify digital skills requirements of jobs. In their work, the authors investigate whether rising technologies are covered in or being incorporated to the set of skills in the ESCO database. Using text mining techniques and based on the lexicon of ‘Industry 4.0’ technologies previously developed in [21], the authors identify rising technologies as those technologies increasingly being referred to in the scientific literature. The technologies obtained from this search are then compared to the 4.0 technologies found in the text description, labels and alternative labels of skills contained in the ESCO database. The authors find that the bulk of the top-80 most frequently cited technologies in the scientific literature are also covered in the most current version of the ESCO database. Moreover, compared to its previous version, the current version of the ESCO database successfully incorporates the technologies that had the fastest growth in citations in the scientific literature.

### The ESCO database

To construct the indicator, we rely on the detailed classification of ESCO. The first version of this classification was released by the European Commission in 2017 and it has been continuously improved since then. We use the latest ESCO version—version v1.1, released in January 2022—that provides information on 3,008 occupations (the occupational pillar) and 13,890 skills/competences and knowledge (the skill pillar). Jointly these two pillars offer insights on which skills are required to perform an occupation.

Each of the 3,008 ESCO occupations is mapped to one of the 436 occupations at the lowest level of the International Standard Classification of Occupations (ISCO-08). For instance, the ESCO occupation ‘ICT security manager’ belongs to the 4-digit occupation ‘Database and network professionals not elsewhere classified’, code 2529, of the ISCO-08 classification. Since the ISCO-08 is hierarchically structured, this hierarchy is also applicable to the ESCO occupations which can then be used to compare results derived from ESCO to other research based on or convertible to the ISCO-08 classification.

For the skill pillar, ESCO provides a semi-hierarchical structure where skills are grouped at multiple levels of detail. Although this structure is not strictly hierarchical as some skills belong to multiple skill groups, it provides useful information on the degree of association between skills, which we use as a starting point for the identification of digital skills from the ESCO database.

One crucial feature of the ESCO database is that skills are mapped to occupations. The database provides information on the skills that are ‘essential’ to an occupation and the skills that are ‘optional’ to it. For instance, the ESCO occupation ‘ICT security manager’ is characterised by 21 ‘essential’ skills (e.g. manage IT security compliances) and 41 ‘optional’ skills (e.g. provide technical documentation).

To simplify notation, the term occupation is used throughout the text to refer to occupations based on the ESCO classification, while the classification name is explicitly indicated when referring to occupations based on the ISCO-08 classification. Likewise to simplify notation, we use the term ‘skills’ to refer to both skills and knowledge in the remainder of this paper.

### Methods

#### The Revealed Comparative Advantage (RCA) statistic

In this paper, we use the Revealed Comparative Advantage (RCA) statistic both in order to measure the degree of dependence of occupations on digital skills and in order to identify terms that are strongly related to digital skills. The method of the RCA was introduced by [22] to analyse the effects of trade liberalisation and it has been since applied in a variety of research fields (e.g. [23–26]), including labour economics [27, 28].

In Eq 1, we present the formula of the RCA statistic used to measure the degree of dependence on digital skills of occupation *j*. To this end, we construct a bipartite network of jobs/occupations (*j* ∈ *J*) and skills (*s* ∈ *S*), where [*I*_*js*_] is the adjacency matrix whose element *I*_*js*_ takes on value 1 if the skill *s* is required to perform occupation *j* and 0 otherwise. We also identify skills that are digital by means of the indicator *D*_*s*_, which takes on value 1 for digital skills. The first line of Eq 1 can be further simplified by noting that $\sum_{s=1}^{S}I_{js}D_{s}$ equals the number of *digital* skills of job *j* ($S_{j}^{D}$) and that $\sum_{s=1}^{S}I_{js}$ equals the *total* number of skills of job *j* (*S*_*j*_)—the degree of *j*, following the network terminology. As shown in the last line of Eq 1, the RCA indicator can also be expressed as the ratio of the share of *digital* skills in occupation *j* (*Dig*_*Share*_*j*_) to the share of *digital* skills in the whole bipartite network of jobs/occupations and skills (*Dig*_*Share*).

A RCA larger than 1 indicates that, relative to all other jobs, occupation *j* is more intensive in the use of digital skills and, thus, it has a revealed comparative advantage in digital skills.

$$RCA_{j}=\frac{\left[\sum_{s=1}^{S}I_{js}D_{s}]/[\sum_{s=1}^{S}I_{js}\right]}{\left[\sum_{j=1}^{J}\sum_{s=1}^{S}I_{js}D_{s}]/[\sum_{j=1}^{J}\sum_{s=1}^{S}I_{js}\right]}$$ (1)

$$\begin{array}{cc} & =\frac{S_{j}^{D}/S_{j}}{\sum_{j=1}^{J}S_{j}^{D}/\sum_{j=1}^{J}S_{j}}\\ & =\frac{Dig\_Share_{j}}{Dig\_Share} \end{array}$$ (2)

Since $\frac{1}{Dig\_Share}$ is constant across occupations, then *RCA*_*j*_ and *Share*_*j*_ are linearly related, and thus, their correlation is 1. For regression analyses, one could use either the *RCA* indicator or directly the share of digital skill of occupations (*Dig*_*Share*_*j*_). Using the RCA definition has the advantage, however, of directly assessing the degree of digitalisation of an occupation relative to the mean digitalisation of occupations in the sample. Because of this, most of the results presented here are based on the RCA indicator.

In the remainder of the paper, we refer to the RCA indicator as the Digital Competencies Indicator (DCI) to measure the digital skill intensity of occupations.

The construction of the DCI indicator entails the identification of the skills that are digital among all skills covered in the ESCO database. In the following section, we describe the steps undertaken in this paper in order to identify digital skills.

#### Identification of digital skills

We use two approaches to identify digital skills from the ESCO database. The first approach employs the hierarchy of skills provided by ESCO and the second approach builds on the first and identifies additional digital skills using Natural Language Processing (NLP).

For the first approach, we start by selecting skill groups from the hierarchy of skills whose name contains the terms ‘computer’, ‘ict’, or ‘digital’. From this exercises we find four skill groups; namely, ‘Information and communication technologies (icts)’, ‘Working with computers’, ‘Working with digital devices and applications’, and ‘Designing ICT systems or applications’. These four groups are the starting point to identify the set of digital skills that we will subsequently use for the definition of jobs/occupations that are intensive in the use of digital skills.

In particular, we define digital skills as any skill belonging to at least one of the four groups. This is done by conducting a downstream crawling of the database that starts at the top level in the hierarchy of a group and continues throughout the whole constellation of skills until no more narrower skills are available.

From this exercise, we identify 918 distinct skills (Table 1). Most of the digital skills belong to ‘Working with computers’, which accounts for 67 per cent of the digital skills identified, followed by ‘Information and communication technologies (icts)’, with 35 per cent. Note that as shown in Table 2, which lists examples of digital skills by digital skills groups, a digital skill can belong to more than one skill group. For instance, ‘penetration testing tool’ belongs to both ‘Working with digital devices and applications’, and ‘Designing ICT systems or applications’. Then, due to duplication of skills across groups, the sum of skills across skills groups (1,019) is larger than the number of distinct digital skills (918).

**Table 1:** Number of skills per digital skill group.

	Number of skills
Information and communication technologies (icts)	322
Working with computers	618
Working with digital devices and applications	46
Designing ICT systems or applications	33
Digital Skills	918

**Table 2:** Examples of digital skills by digital skill groups.

Information and communication technologies (icts)	
SQL Server Integration Services	database quality standards
Windows Phone	Puppet (tools for software configuration management)
Maltego	SAS language
Working with computers	
edit negatives	adapt to changes in forestry
operate railway control panels	types of wire mesh
advanced driver assistant systems	operate ride panel
Working with digital devices and applications	
security panels	Schoology
Pascal (computer programming)	penetration testing tool
Capture One	Litmos
Designing ICT systems or applications	
KDevelop	software metrics
tools for ICT test automation	Absorb (learning management systems)
data protection	penetration testing tool

Note: randomly selected skills per skill group.

The second approach used to identify digital skills takes advantage of the wealth of information attached to individual skills in the ESCO database. As shown in Table 3, besides the label of the skills, the ESCO database also includes alternative labels and a description of the skill. In this approach, we first gather text data from the label, alternative labels and the description for all skills and for digital skills as defined in the first approach described above. We then calculate the frequency of words for all skills and compare it with the frequency of words for digital skills by means of the RCA statistic.

**Table 3:** Examples of text for selected skills.

Information and communication technologies (icts)	
*Label*: SQL Server Integration Services	*Description*:
*Alternative labels*: Microsoft SQL Server Integration Services, Microsoft SSIS, SSIS.	The computer program SQL Server Integration Services is a tool for integration of information from multiple applications, created and maintained by organisations, into one consistent and transparent data structure, developed by the software company Microsoft.
Working with computers	
*Label*: edit negatives	*Description*:
*Alternative labels*: alter negatives, editing of negatives, retouch negatives, edit the negatives.	Use different software products and techniques to process photographic negatives and adapt the images to the desired specifications.
Working with digital devices and applications	
*Label*: security panels	*Description*:
*Alternative labels*: security panel components, wire contact points, motherboard, transformer, security system panels, processor, components of security panel.	The internal logic of the security panel, where security sensors send their data for processing. The different components of the panel, such as the wire contact points, motherboard and transformer.
Designing ICT systems or applications	
*Label*: KDevelop	*Description*:
*Alternative labels*: KDevelop 4.7.0, KDevelop 4.6.0, KDevelop 4.0.0, KDevelop 5.0.0.	The computer program KDevelop is a suite of software development tools for writing programs, such as compiler, debugger, code editor, code highlights, packaged in a unified user interface. It is developed by the software community KDE.

Note: randomly selected skills per skill group.

**Fig 1:**
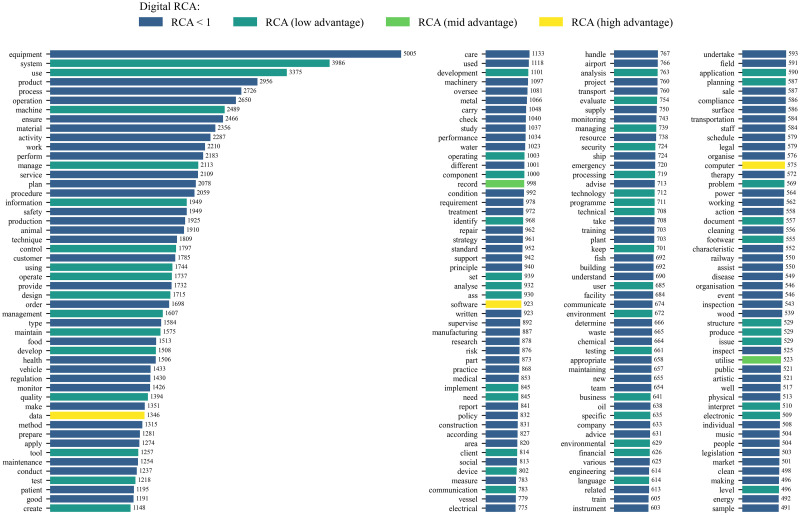
Text analysis: Most frequent terms and RCA of terms. Note 1: term counts based on the label, alternative labels and the description of skills. Note 2: a RCA < 1 means that the term is less frequent among digital skills relative to all skills. Thus the term has a relative comparative ‘disadvantage’ describing digital skills. RCA (low advantage) for digital RCA between 1 and 5; RCA (mid advantage) for digital RCA between 5 and 10; and RCA (high advantage) for digital RCA greater than 10. Note 3: for the text analysis, we first tokenise the text, we then discard punctuation marks and stop-words such as ‘from’, ‘the’, and ‘further’, and finally, we apply a lemmatisation using Python NLTK package for Natural Language Processing (NLP).

Fig 1 shows the 200 most frequent words for all skills obtained from this exercise. With 5,005 references, ‘equipment’ is the most cited word followed by ‘system’ with almost 4,000 mentions. The figure also categorises words by their digital RCA. In this context, the RCA describes the importance of a word in the texts for digital skills relative to its importance in the texts for all skills. Among the most frequent terms, only ‘data’, ‘software’ and ‘computer’ have a high value of digital RCA.

We then select a set of words deemed to be suitable for describing digital skills based on their RCA value and their frequency in the text. For the threshold criterion, we use the RCA of the term ‘digital’ as a benchmark. The term ‘digital’ has a RCA that is at the 94^*th*^ percentile of all digital RCA values in the database. Therefore, we only retain terms with RCA values at the 94^*th*^ percentile or above.

In the final step of this approach, we identify a set of additional digital skills by matching skills’ labels and alternative labels to the set of 20 selected digital terms. From this matching, we identify 233 new digital skills that we use along with the digital skills identified in the first approach in order to construct the digital competencies indicators. As shown in Table 4, ‘ICT quality policy’, ‘audio technology’, and ‘digital printing’ are examples of the new digital skills that were not accounted for by the first approach.

**Table 4:** Examples of new digital skills through NLP.

ICT quality policy	aerospace engineering
analyse ICT technical proposals	audio technology
create concept of digital game	digital printing
evaluate cost of software products	interview animal owners on animals’ conditions
keep up-to-date to computer trends	locate microchip in animals
oversee development of software	product data management
provide software testing documentation	read broadcast programming
read technical datasheet	respond to incidents in cloud
review meteorological forecast data	tend spring making machine

Note 1: random selection of digital skills identified through terms matching.

Note 2: the 20 matching terms (ordered from most to less frequent) are data, software, computer, digital, ict, database, programming, cad, cloud, cnc, algorithm, computer-aided, server, integration, virtual, query, scanner, microsoft, aeronautical, and configuration.

#### Digital competencies indicator

Using the list of digital skills defined in the previous section alongside the mapping of occupations and skills provided in the ESCO database, we construct four different versions of the DCI indicator. The versions differ in two respects: (i) either solely *essential* skills or both *optional* and *essential* skills are used; (ii) digital skills are identified *solely* based on the skill groups provided by ESCO or they are *additionally* identified by employing NLP and by matching digital terms to skills’ texts. This leaves us with four different combinations:

- Option 1—Essential skills and digital skills classified according to skill groups
- Option 2—Essential plus optional skills and digital skills classified according to skill groups
- Option 3—Essential skills and digital skills classified according to skill groups plus additional terms
- Option 4—Essential plus optional skills and plus digital skills classified according to skill groups plus additional terms

### Results

#### DCI by ESCO occupations

**Fig 2:**
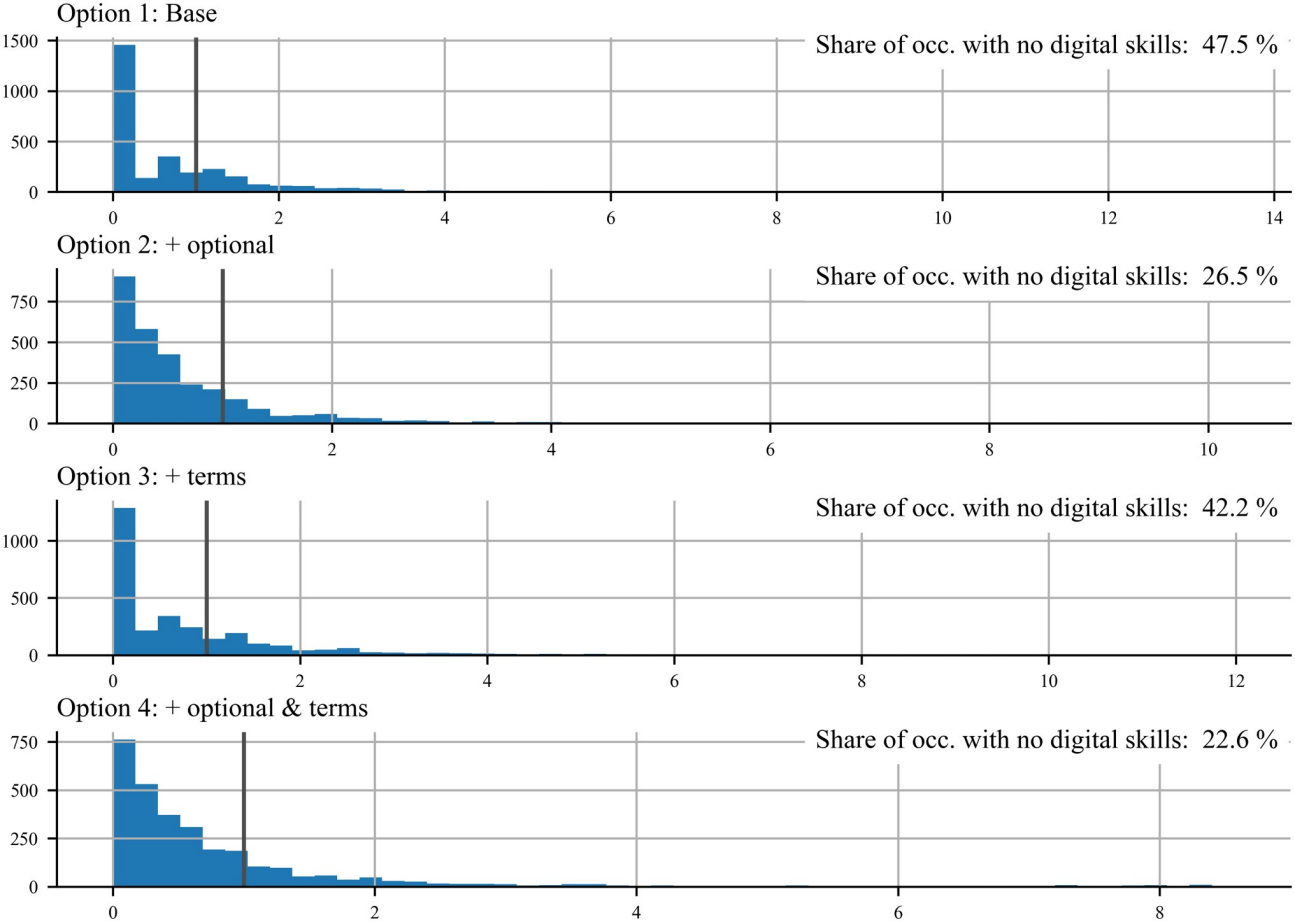
Distribution of DCI indicators across occupations. Note: we have added a line at value one in order to visually distinguish occupation groups with a revealed comparative advantage from those with a revealed comparative disadvantage.

**Fig 3:**
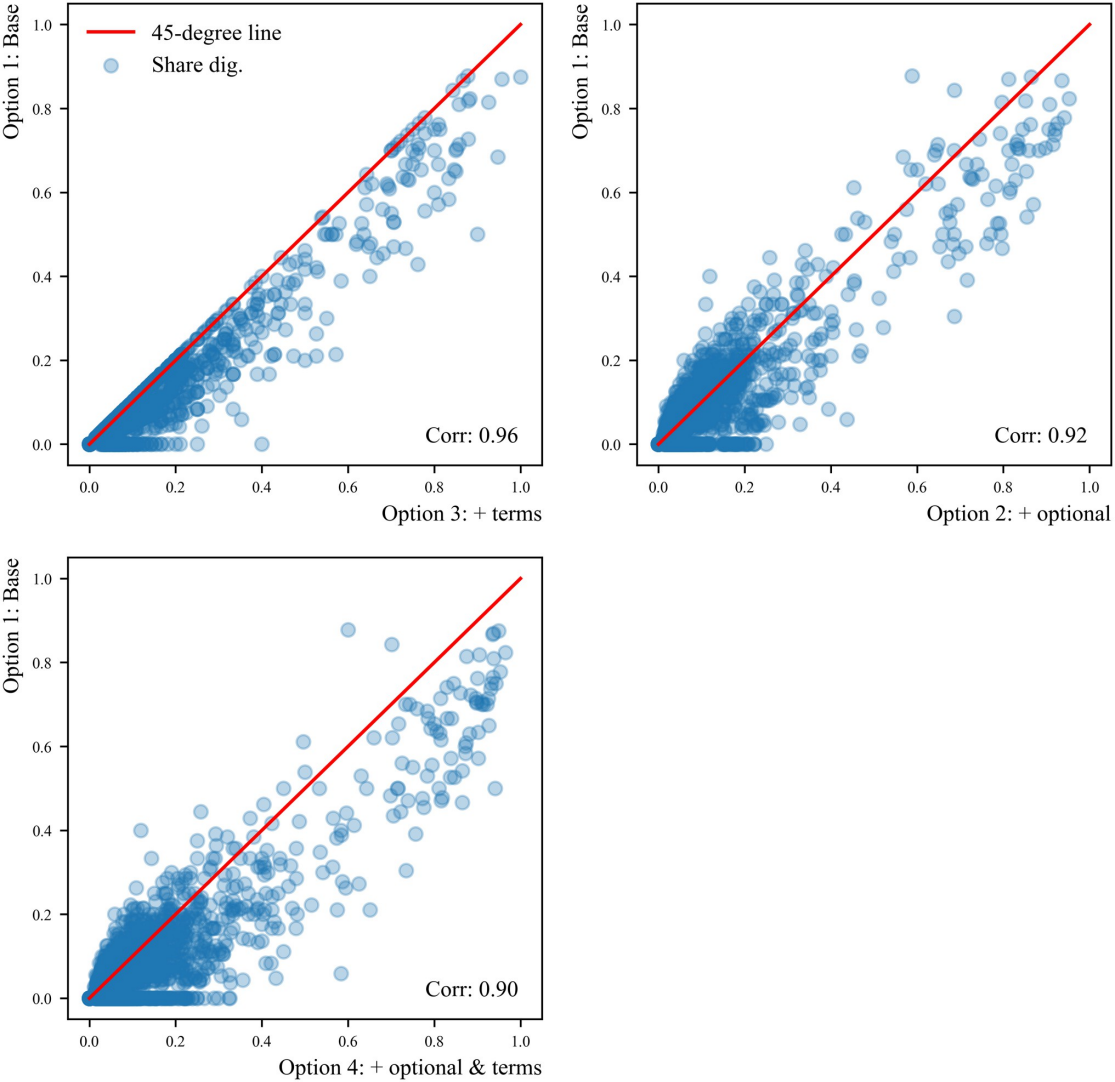
Scatter plots: Share of digital skills per occupation. Note: we have added the 45 degree line to facilitate the visual comparison between versions.

The results on the four versions of the DCI indicator are presented in Table 5, Figs 2 and 3. Table 5 shows some simple statistics of the four DCI indicators and of the main inputs used in their construction. Fig 2 plots the distribution of the DCI across occupations and Fig 3 presents the scatter plots of the share of digital skills across occupations in the base version (Option 1) against the share of digital skills in versions 2, 3 and 4.

**Table 5:** DCI versions: Means, medians, maximum and minimum values across occupations.

	DCI				Share of dig. skills			
	max	mean	median	min	max	mean	median	min
Option 1: Base	13.521	0.977	0.428	0.000	0.878	0.063	0.028	0.000
Option 2: + optional	10.237	0.824	0.413	0.000	0.953	0.077	0.038	0.000
Option 3: + terms	11.984	0.973	0.479	0.000	1.000	0.081	0.040	0.000
Option 4: + optional & terms	8.570	0.835	0.423	0.000	0.965	0.094	0.048	0.000
	Number of dig. skills				Number of skills			
	max	mean	median	min	max	mean	median	min
Option 1: Base	44.000	1.405	1.000	0.000	133.000	21.648	19.000	2.000
Option 2: + optional	99.000	3.833	1.000	0.000	340.000	41.174	36.000	4.000
Option 3: + terms	46.000	1.806	1.000	0.000	133.000	21.648	19.000	2.000
Option 4: + optional & terms	106.000	4.635	2.000	0.000	340.000	41.174	36.000	4.000

The minimum value of the DCI indicator is zero across versions (Table 5). Indeed, the share of occupations without digital skills in the base version is large, but it halves as we add digital skills from the NLP analysis and optional skills in the construction of the indicator. As such, the share of occupations without digital skills declines from 48 per cent in the base version (Option 1) to 23 per cent in the full version (Option 4) as shown in Fig 2. The reduction in the share of occupations without digital skills is mainly driven by the inclusion of optional skills. Most probably because by increasing the number of skills relevant to one occupation, one increases as well the likelihood of an occupation of having at least one skill being digital.

The mean value of the DCI indicator is not of interest by itself because the RCA statistic is a relative measure whose mean value should always be around one. The mean value would be exactly one if all occupations had the same number of skills in the database. The distance between the indicators’ mean and their median provides us, on the other hand, with valuable information on how skewed the distribution of the DCI is across occupations. The median values are around one half of the mean values of the indicator across versions. This means that the distribution of the indicator has a long tail to the right of the distribution, which can be observed in Fig 2. The tail is the largest for the base option that has the largest maximum DCI value (13.5). Adding both additional digital skills based on the NLP analysis and optional skills shortens the tail.

Option 3, which identifies a larger number of digital skills, unsurprisingly increases the share of digital skills per occupation to 8.1 per cent, up from 6.3 per cent in the base version. More surprisingly perhaps is that the share of digital skills also increases as we add optional skills (Option 2). This indicates in turn that optional skills are relatively more digital than essential skills, maybe because they refer to a more specialised type of skills compared to essential skills that might be relatively more generic. Also worth mentioning is that, compared to the base version, the average number of skills per occupation almost doubles when we add skills that are optional, from 22 in the base version to 41 in Option 3.

Because of the nature of the indicator one should expect a monotonic relationship between the number of occupations and their digital intensity, with the number of occupations associated with a particular level of digital intensity declining as the digital intensity increases. In other words, one should expect a large number of occupations with little digital competencies requirements and fewer and fewer occupations as the requirement on digital competencies increases. As shown in Fig 2, in all versions the group of occupations with the largest frequency are those with the lowest digital intensity (i.e. lowest DCI). However, the relationship between frequency and digital intensity is not monotonic in the base version (Option 1). Monotonicity improves when we add optional skills in the construction of the DCI (Option 2), for this version the number of occupations smoothly declines as the DCI indicator increases. Monotonicity also improves, although to a lesser extent, when we add digital skills from the NLP analysis (Option 3).

As shown by the scatter plots in Fig 3, all versions are highly correlated and the correlation between versions is never below 0.9. The highest correlation is found between the base option and Option 3, adding digital skills based on NLP analysis, while the lowest is found between the base option and the full indicator (Option 4). A 45 degree line has been added in the scatter plots in order to facilitate the comparison between DCI versions. It can be seen that, relative to the base version, Option 3 increases the share of digital skills at any level of digital intensity. Including optional skills (Option 2), on the other hand, increases the share of digital skills for some occupations, while decreasing it for others.

Compared to the base option, both the inclusion of additional digital skills through the NLP analysis and the inclusion of optional skills in the construction of the indicator bring about improvements. Through the NLP analysis, we are able to identify skills such as ‘ICT quality policy’, ‘audio technology’, and ‘digital printing’, which, although clearly related to digital competencies, were not covered in any of the digital skills groups of the ESCO database. Using optional skills further taps into the wealth of information provided in the ESCO database. By almost doubling the number of skills linked to occupations, the use of optional skills resulted in a smoother distribution of occupations over the DCI values. Adding optional skills requires, however, taking an arbitrary decision on the way in which optional skills are weighted in the construction of the final indicator. By giving essential and optional skills the same weight, we have presented the most extreme way of including optional skills in this paper. Any reduction in the importance assigned to optional skills in the construction of the DCI indicator will result in indicators being closer to those in the base option. For the sake of brevity, we thus retain Option 3, which adds digital skills based on the NLP, for the remaining analyses in this paper.

So far, we have presented the results of the DCI indicator at the lowest level of the classification of occupations in the ESCO database. One important feature of our indicator, however, is that it can be easily mapped to occupations according to the ISCO classification. This allows researchers to apply the indicator in analyses on employment, earnings and social exclusion based on surveys adhering to the ISCO classification, such as the European Labour Force Survey (EU-LFS), the survey on Income and Living Conditions (EU-SILC) and the European Structure of Earnings Survey (SES), among others.

We thus present in the following section additional results on the DCI indicator based on Option 3 and aggregated according to the latest version of the ISCO classification (ISCO-08).

#### DCI by ISCO 2-digit groups

In this section we analyse the results of the DCI indicator aggregated at the 1-digit level and the 2-digit level of the ISCO-08 classification, referred to as major and submajor groups, respectively.

**Fig 4:**
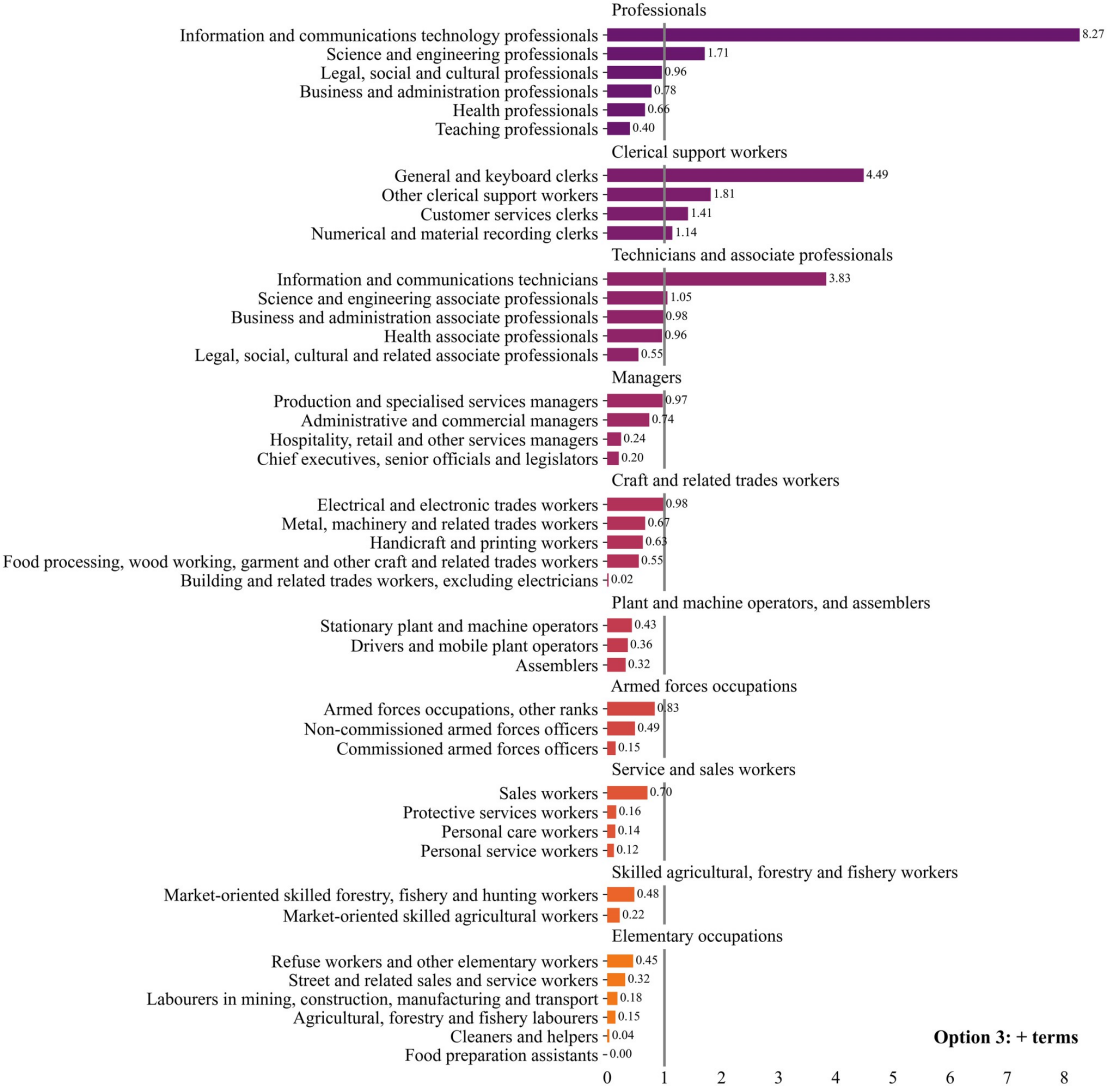
Mean values of DCI by 2-digit ISCO code. Note 1: we have added a line at value one in order to visually distinguish occupation groups with a revealed comparative advantage from those with a revealed comparative disadvantage. Note 2: the mean values of the DCI by major groups are as follows; Professionals (1.66), Clerical support workers (1.50), Technicians and associate professionals (1.03), Managers (0.72), Craft and related trades workers (0.57), Plant and machine operators, and assemblers (0.41), Armed forces occupations (0.37), Skilled agricultural, forestry and fishery workers (0.32), Service and sales workers (0.34), and Elementary occupations (0.20).

Fig 4 plots the mean values of the DCI indicator aggregated at the 2-digit level of the ISCO classification. The 2-digit groups are in turn clustered by 1-digit groups and plotted in descending order according to the mean value of the indicator. To facilitate the visual identification of the occupations with a revealed comparative advantage in digital skills, we have drawn a line at value one.

Among major groups, only three out of the ten major groups have a revealed comparative advantage in digital skills; ‘Professionals’ (1.66) have the highest mean DCI value, followed by ‘Clerical support workers’ (1.50) and ‘Technicians and associate professionals’ (1.03). All the other major groups have DCI mean values below one, with ‘Elementary occupations’ (0.20) registering the lowest score.

Among 2-digit groups, only 8 out of the 42 submajor groups have DCI values over one, and thus have a revealed comparative advantage in digital skills. ‘Information and communications technology professionals’ (8.27) scores the highest, followed by ‘Information and communications technicians’ (3.83), and ‘General and keyboard clerks’ (4.49). At the other end of the spectrum, we find ‘Cleaners and helpers’ (0.04), ‘Building and related trades workers’ (0.02), and ‘Food preparation assistants’ (0.00) with the lowest digital skill requirements.

The inspection at the 2-digit level also reveals a large dispersion of the DCI values within some of the major groups. We find, for instance, that for the major groups of ‘Professionals’ and of ‘Technicians and associate professionals’ not all of their submajor groups have on average a revealed comparative advantage in digital skills. All the other major groups are relatively more homogeneous, in that either all of their submajor groups have average values below one or all of them have DCI values above one.

**Fig 5:**
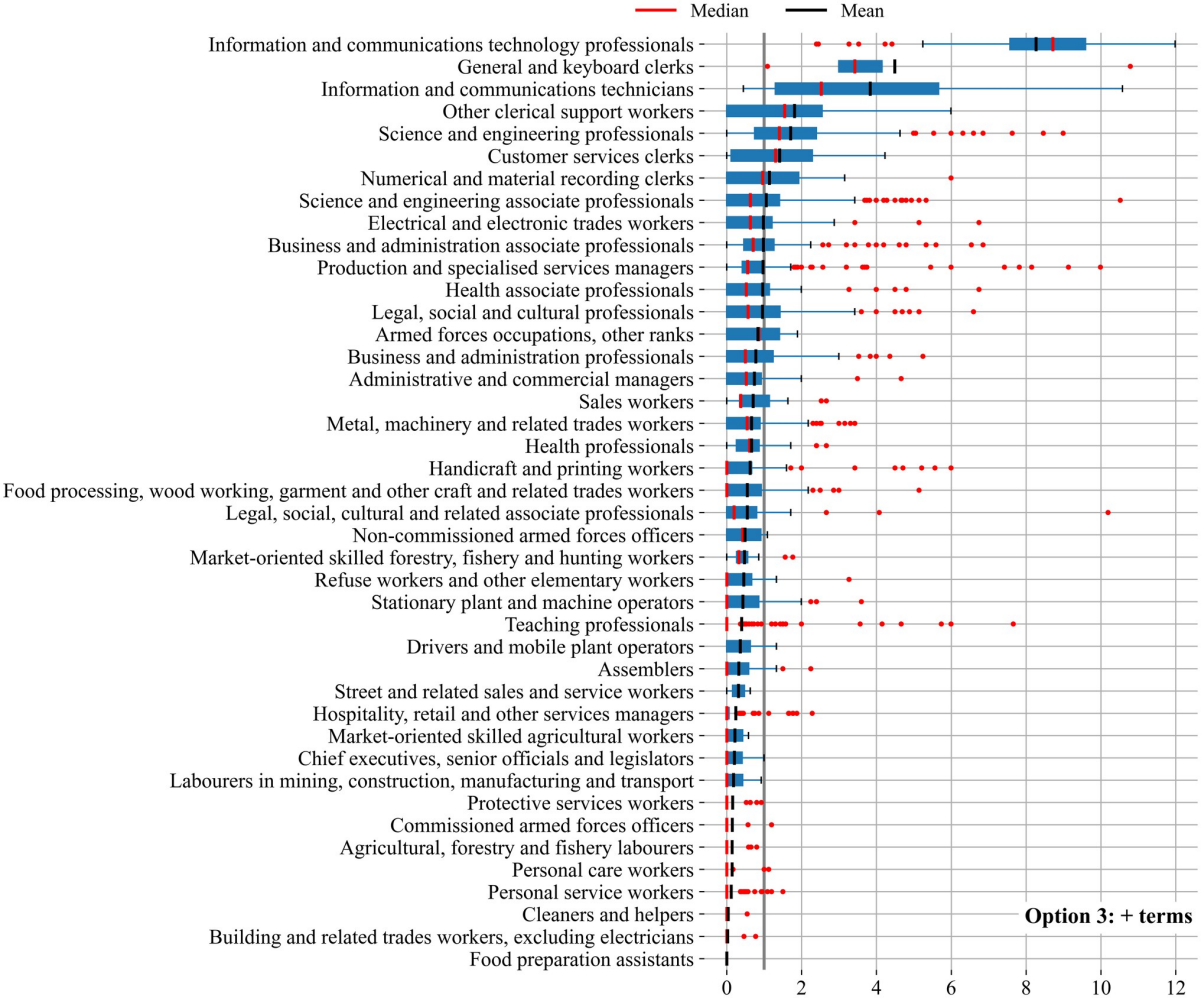
Boxplot of DCI by occupations: 2-digit ISCO code. Note: we have added a line at value one in order to visually distinguish occupation groups with a revealed comparative advantage from those with a revealed comparative disadvantage.

Fig 5 shows the boxplot of the DCI values of occupations grouped by ISCO submajor groups, where red points refer to ESCO occupations and boxes to ISCO submajor groups. This figure highlights that even within submajor groups there is still important variation in the DCI value across occupations. While there are some submajor groups having either all occupations with DCI values below one or all occupations with values above one, the majority of submajor groups, such as those in the middle, consist of a mixture of occupations, some of them having DCI values below one, while others above one.

We think that the 2-digit aggregation of the DCI already provides useful insights into the degree of digitalisation of occupations. However, given the still considerable dispersion of DCI scores within submajor groups, if feasible, researchers should try to employ the DCI indicator at the lowest possible level of aggregation of the ISCO classification.

## Comparing the DCI to a task-based indicator

One might question the relevance of using information from occupational dictionaries if there are significant country differences in terms of tasks carried out at work. We therefore turn our attention to the real labour market and compare our indicator with a task-based approach. In this way, we account for the fact that peoples’ self-reported tasks may differ from the skill requirements of occupations derived from a dictionary of occupations and we provide an example of how the DCI can be used in combination with other databases.

In this section, we use information on ICT use at work from the PIAAC survey, which has become a popular source for researchers interested in the task-content of jobs and which has also been used to construct task indices based on questions on the frequency of using ICT at work (e.g. [8, 9, 18]). Moreover, the PIAAC survey provides population weights and information on occupations based on the ISCO-08 classification. Its employment estimates by occupations can thus be applied to the DCI, thereby helping to gain better insights on the employment structure in an economy with a focus on digital skill requirements.

PIAAC is therefore an ideal source to illustrate the benefits and potential applications of using the ESCO-based DCI alongside labour market surveys. In addition, combining DCI and PIAAC helps to highlight differences between the two different approaches to measure digital skills within occupations—the first one relying on tasks captured in survey data and the second one exploiting information on skills in a dictionary of occupations.

### PIAAC data

The PIAAC survey has been conducted in more than 40 OECD countries and provides information on the frequency of tasks carried out at work, among others. In each participating country, a representative sample of around 5,000 individuals aged 16 to 64 years is interviewed in order to collect data on skill use at work and in everyday life. The survey also provides information on sociodemographic background characteristics, working situation and proficiency within three competency domains (literacy, numeracy and problem-solving).

For the purpose of this paper, we use data from PIAAC on a set of 13 European countries that provide occupations on the ISCO-08 4-digit level. These countries are Belgium, Czech Republic, Denmark, Spain, France, Great Britain, Italy, Netherlands, Poland, Slovakia, Greece, Lithuania and Slovenia. The latest data collection took place in 2011/2012, so all PIAAC-based results refer to the task content of occupations from ten years ago.

We restrict the sample to respondents that were employed according to the international definition (having paid work in the week prior to the date of the interview) leaving us with a total of 43,975 observations, where the number of observations per country ranges from 2,288 in Greece to 4,697 in Denmark.

### PIAAC indicator

We focus on the questions in PIAAC related to the frequency of tasks that involve the use of the internet and/or a computer (see Table 6).

**Table 6:** ICT use at work variables in PIAAC.

*In your current job, how often do you*….	PIAAC Code
…use email?	G_Q05a
…use the internet in order to better understand issues related to your work?	G_Q05c
…conduct transactions on the internet, e.g., buying or selling products or services, or banking?	G_Q05d
…use spreadsheet software, e.g., Excel?	G_Q05e
…use a word processor, e.g., Word?	G_Q05f
…use a programming language to program or write computer code?	G_Q05g
…participate in real-time discussions on the internet, e.g., online conferences, or chat groups?	G_Q05h

The responses are re-scaled to range from 0 to 1 where 0 denotes ‘never’, 0.25 denotes ‘less than once a month’, 0.5 denotes ‘less than once a week but at least once a month’, 0.75 denotes ‘at least once a week but not every day’ and 1 denotes ‘every day’. Based on these variables in PIAAC (denoted as *g*_*ij*_, *j* ∈ *J* where *J* refers to the set of ICT tasks), the average ICT task use score is calculated as arithmetic mean for each survey participant *i* that was employed at the time of the interview, i.e. $ict_{i}=\frac{\sum_{j\in J}g_{ij}}{J}$. Since our goal is to derive indicators that are representative of the workforce at the lowest possible level of the ISCO classification, we calculate the weighted means (using the PIAAC final weights) by ISCO-08 4-digit occupation for each of the European countries in PIAAC providing this information. To derive the European indicator, we take the simple mean across all countries. In the following, we refer to the individual country indicators as ‘DCI PIAAC’ and to the average across countries as the ‘European DCI PIAAC’.

To merge the DCI Option 3 with the PIAAC data, the DCI index is first aggregated at the ISCO-08 4-digit level by taking the mean across occupations. Each respondent in the PIAAC data is then assigned a DCI value based on their ISCO-08 4-digit occupation. This allows us to analyse the degree of digitalisation of the employed population for our country sample.

### Results

As a first step, we use the DCI PIAAC at the ISCO-08 4-digit level to see whether the digital task content of occupations differs across European countries. A comparison of the occupation ranks in each country with the occupation ranks based on the European average shows that they are highly correlated (Table 7) with the Spearman’s correlation coefficients ranging from 0.83 to 0.89.

**Table 7:** Correlation between countries’ and the European DCI PIAAC.

Country	Spearman’s *ρ*
BEL	0.859
CZE	0.843
DNK	0.843
ESP	0.832
FRA	0.886
GBR	0.887
GRC	0.840
ITA	0.839
LTU	0.839
NLD	0.885
POL	0.859
SVK	0.858
SVN	0.867

This implies that the ICT-task profiles of workers in Europe are very similar within ISCO-08 4-digit occupations and this can further be interpreted as evidence that the ESCO DCI can be applied across countries. Hence, for the remainder of this section we use the European DCI PIAAC and compare it to the DCI based on ESCO to show the differences between measuring digital skill intensity using a task-based approach and a dictionary approach.

A first hint that these differences matter can be derived from the rank correlations between the DCI PIAAC and DCI Option 3. The correlation coefficient suggests that there is a positive relationship between the two indicators, but Spearman’s *ρ* is comparatively low at 0.53.

**Fig 6:**
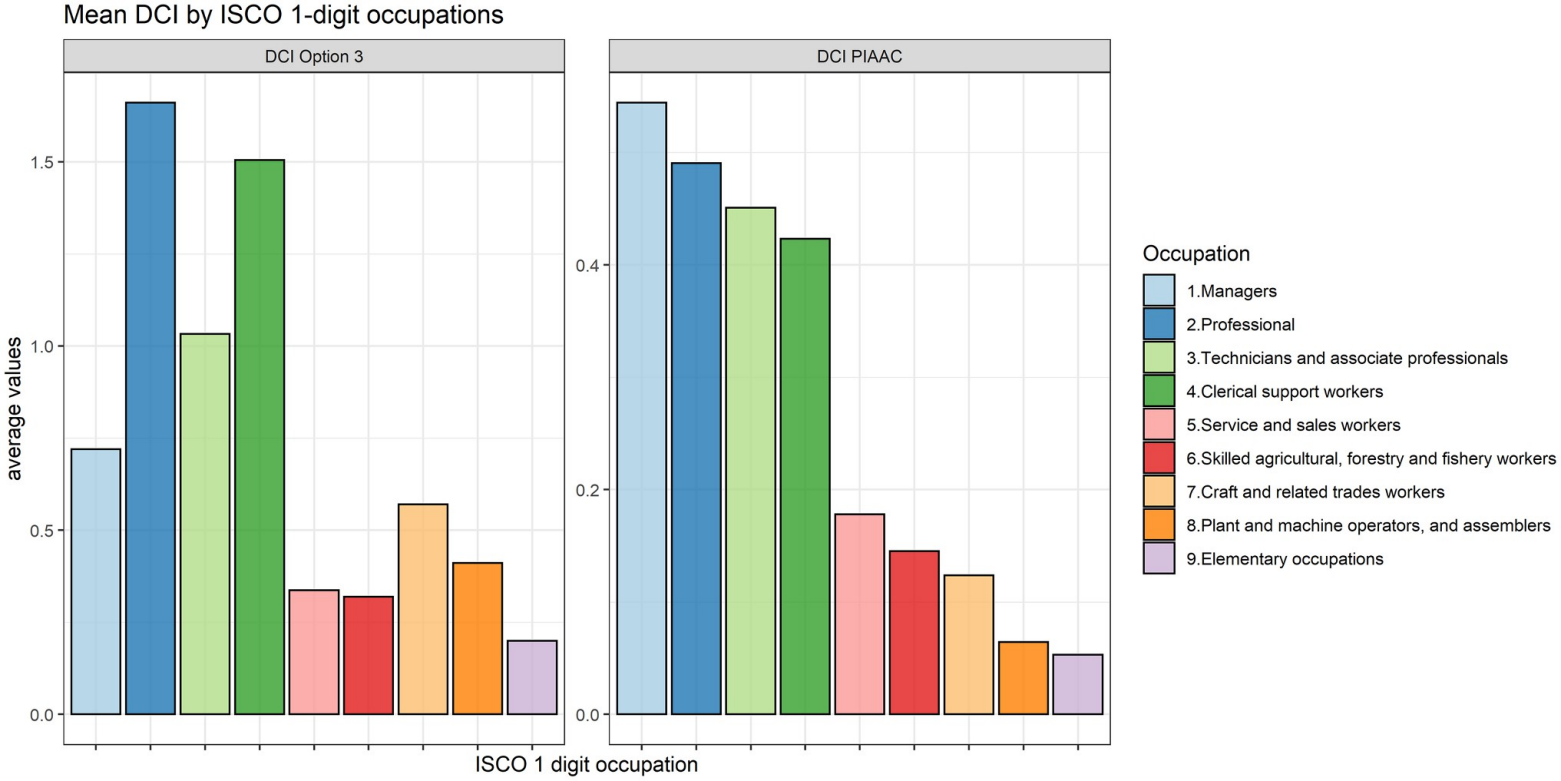
Mean values of DCI by 1-digit ISCO-08 code. Note: the overall occupation means for the PIAAC indicator are based on country means, which are calculated using the final weights provided in PIAAC.

Fig 6 further compares the two indicators, by plotting the mean values of DCI Option 3 and DCI PIAAC at the ISCO-08 1-digit level. The height of the bars shows that the DCI PIAAC (right facet) decreases with the occupational skill level whereas this is not the case for DCI Option 3. For the latter, as can be seen from the left facet of Fig 6, the highest average can be found for ‘Professionals’, followed by ‘Clerical Support Workers’ and ‘Technicians and associated professionals’. The difference between the two indicators is particularly evident for ‘Managers’. For this group the average DCI PIAAC is the highest, while the mean for the DCI Option 3 ranks only fourth and it is closer to the average value for ‘Craft and related trade workers’ and ‘Plant and machine operators’ than to that of ‘Professionals’. This implies that, firstly, the DCI Option 3 is less susceptible to being confounded with the skill level of an occupation. Secondly, it suggests that the DCI Option 3 conveys different information on digital skill requirements compared to what is captured by the DCI PIAAC, which relies on the frequency of ICT use at work.

**Fig 7:**
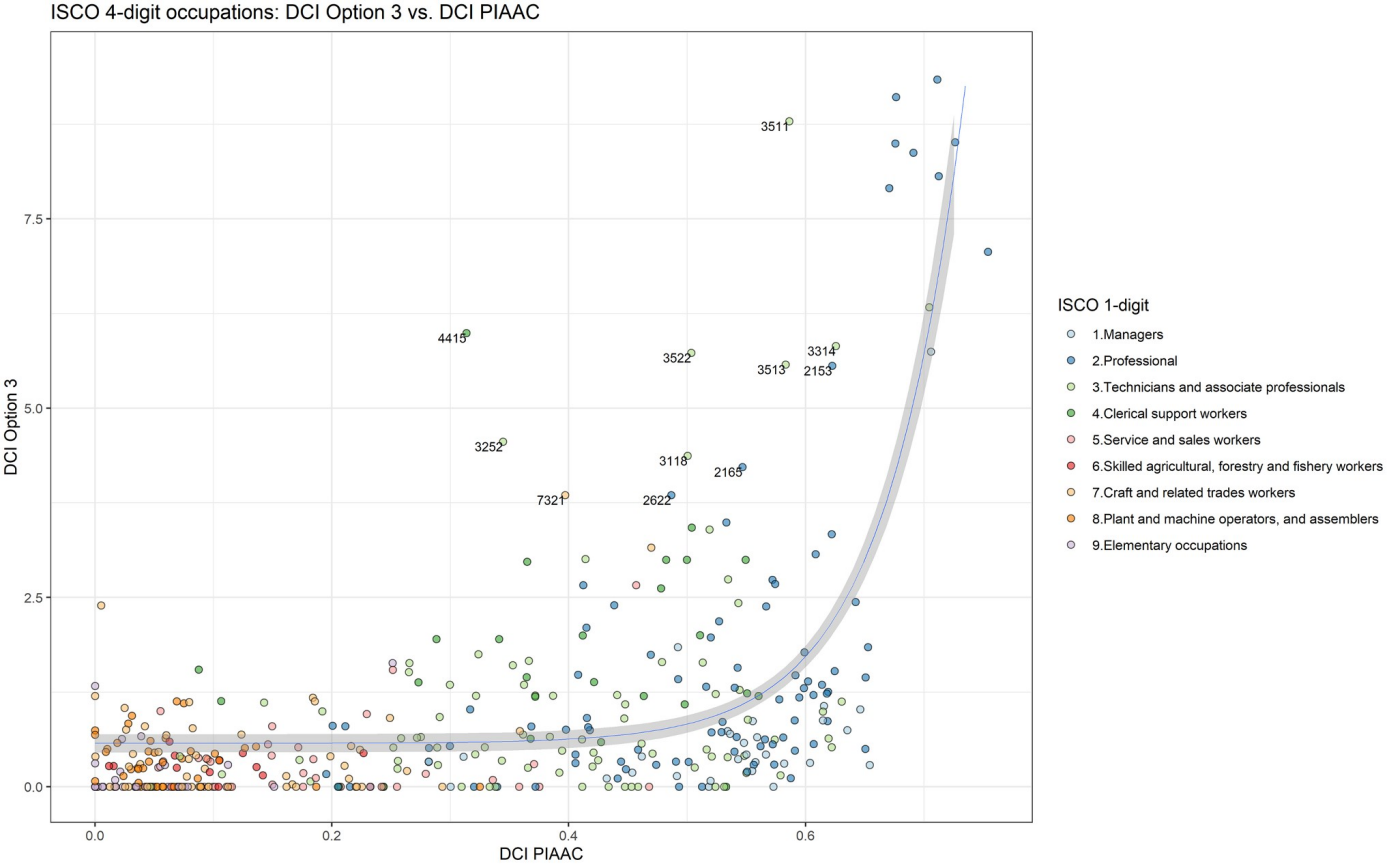
Scatter plot of Option 3 and DCI PIAAC: ISCO-08 4-digit occupations. Note: the labelled occupations indicate those that have a very high ESCO-based DCI compared to the PIAAC-based DCI such as ‘Information and Communications Technology Operations Technicians’ (3511), ‘Filing or Copying Clerks’ (4415), ‘Telecommunications Engineering Technicians’ (3522), or ‘Pre-press Technicians’ (7321).

The relationship between the DCI PIAAC and DCI Option 3 is further illustrated in Fig 7. The DCI Option 3 is plotted against the DCI PIAAC on the ISCO-08 4-digit level and colours indicate the ISCO-08 1-digit submajor group as a proxy of skill level. Two key insights can be taken away from this scatter plot. First, the DCI Option 3 highlights the existence of highly specialised digital occupations, which are clustered at the top right corner of the scatter plot. Second, DCI Option 3 identifies digital occupations that survey data on reported tasks fail to capture. For example, the occupations ‘Information and Communications Technology Operations Technicians’ (3511), ‘Filing or Copying Clerks’ (4415), ‘Telecommunications Engineering Technicians’ (3522) are among the top 20 digital occupations based on DCI Option 3, whereas according to PIAAC, they are located in the (lower) midfield.

**Fig 8:**
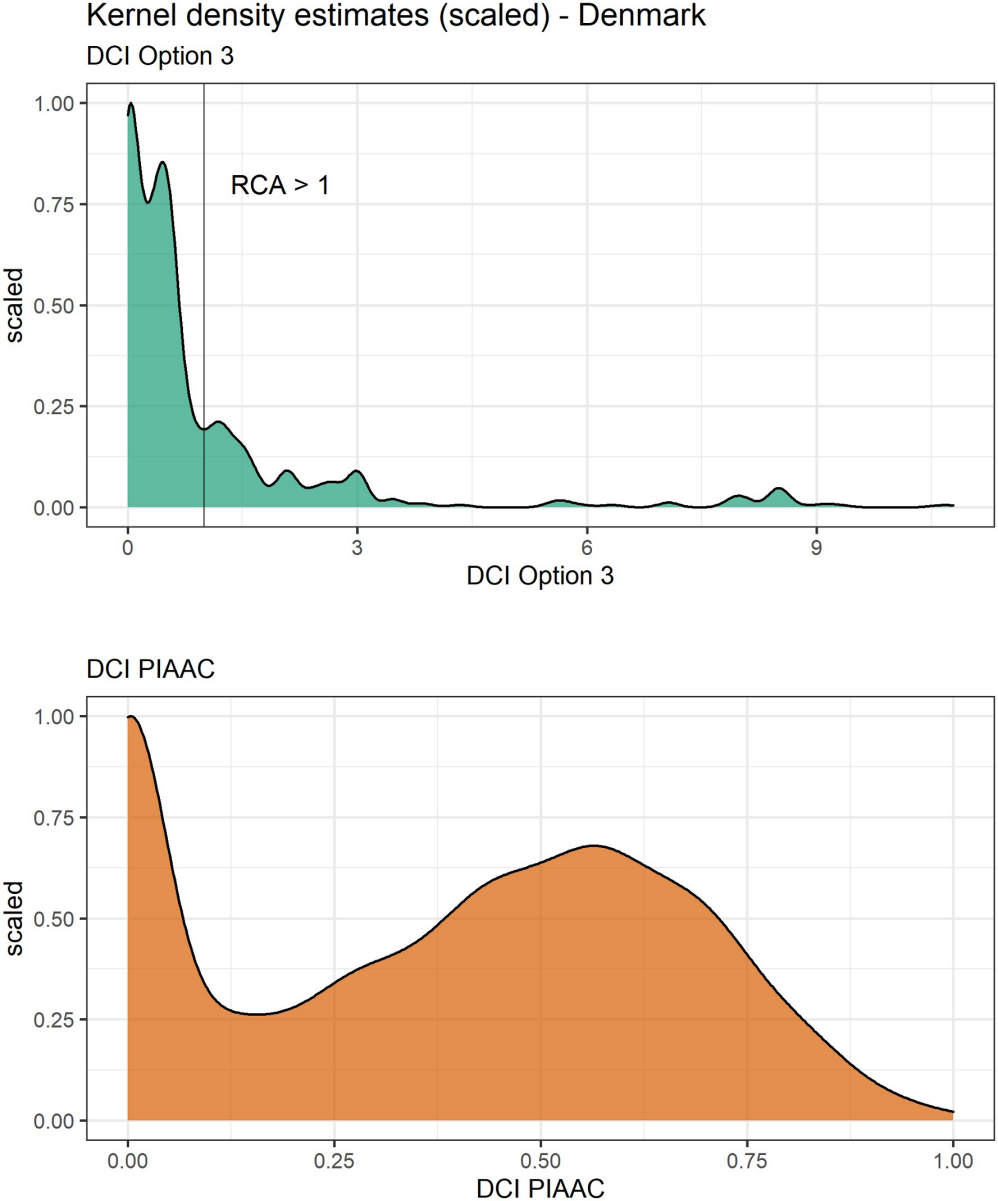
Kernel density estimates: DCI Option 3 and DCI PIAAC in Denmark. Note: the vertical line in the upper panel denotes a RCA of one to visually distinguish between individuals’ occupations with a revealed comparative advantage from those with a revealed comparative disadvantage.

Finally, we single out Denmark, the country with the highest number of observations in our sample, to illustrate how the distributions of the DCI PIAAC and DCI Option 3 differ in the employed population. Fig 8 plots the kernel density estimates (scaled to a maximum of one) using a Gaussian kernel. From this smoothed histogram, we see for DCI Option 3 (upper panel) that the mass of the distribution can be found at the lower end of the density with a very long tail at the upper end resulting from the existence of some highly specialised digitalised occupations. Similarly, the density plot for the DCI PIAAC has a peak at the lower end of the density. However, there is a second peak around the middle of the distribution. This further emphasises that the DCI PIAAC captures rather generic ICT tasks that are used across many occupations and is therefore less suitable to capture nuanced differences between occupations.

In summary, we show that the DCI based on ESCO can easily be applied to other data sources due to its mapping to ISCO-08 4-digit occupations. The comparison with PIAAC further highlights that while the self-reported frequency of carrying out ICT tasks at work can primarily be used to describe typical white-collar office work, it fails to identify occupations that are highly digitalised such as ‘Telecommunications Engineering Technicians’. The ESCO-indicator on the other hand, seems to be reasonably good at differentiating between different degrees of digital specialisation of occupations and it is somehow independent from the skill level (typically proxied by the ISCO-08 1-digit occupations).

## Conclusion

We introduced a novel method to assess the digitalisation of occupations based on skills as opposed to approaches based on tasks. To this end, we use the detailed ESCO taxonomy and employ network analysis methods together with NLP to construct four versions of a digital competencies indicator. Our methods expand on the work presented in [16] by increasing the number of digital skills covered and by constructing our indicators at the lowest level of the ESCO hierarchy of occupations. Since our indicators can be mapped to the ISCO-08 classification, which is a standard classification of occupations, it can be easily applied to labour market survey data. This might be particularly useful as survey data usually only provide information on occupations and industries but not on the requirements of skills at work.

Perhaps one important limitation of the ESCO database is that it does not distinguish between the degree of specialisation and complexity of the skills. Our comparison with the PIAAC based indicator shows, however, that our indicator fares better at describing more complex and higher digitalised occupations, while the PIAAC indicator mainly describes typical white-collar office tasks.

We also find a large dispersion of the value of the indicator within occupational groups, highlighting the importance of using granular data in order to properly identify digital skill requirements of jobs. At the same time, this suggests that, whenever possible, the indicator should be applied at the lowest level of the occupational hierarchy. Finally, the DCI is relatively less susceptible of being confounded with the skill level of an occupation relative to the PIAAC indicator.

## Supporting information

S1 File (XLSX)
